# Supplementary material for: Predicting water status, growth and yield of tomato under different irrigation regimes using the RGB image indices and artificial neural network model
Source: PLoS One. 2026 Apr 30;21(4):e0346503. doi: 10.1371/journal.pone.0346503 (PMC13132235; doi:10.1371/journal.pone.0346503)
Supplement: Attachment — Submitted filename: Comments-3.docx [file pone.0346503.s002.docx]

Predicting water status, growth and yield of tomato under different irrigation regimes using the RGB image indices and artificial neural network model

**Comments to the Author**

**1. Is the manuscript technically sound, and do the data support the conclusions?**

The manuscript must describe a technically sound piece of scientific research with data that supports the conclusions. Experiments must have been conducted rigorously, with appropriate controls, replication, and sample sizes. The conclusions must be drawn appropriately based on the data presented.

Reviewer #1: Yes

Reviewer #2: Yes

**2. Has the statistical analysis been performed appropriately and rigorously?**

Reviewer #1: Yes

Reviewer #2: Yes

**3. Have the authors made all data underlying the findings in their manuscript fully available?**

The PLOS Data policy requires authors to make all data underlying the findings described in their manuscript fully available without restriction, with rare exception (please refer to the Data Availability Statement in the manuscript PDF file). The data should be provided as part of the manuscript or its supporting information, or deposited to a public repository. For example, in addition to summary statistics, the data points behind means, medians and variance measures should be available. If there are restrictions on publicly sharing data—e.g. participant privacy or use of data from a third party—those must be specified.

Reviewer #1: No

Reviewer #2: Yes

**4. Is the manuscript presented in an intelligible fashion and written in standard English?**

PLOS ONE does not copyedit accepted manuscripts, so the language in submitted articles must be clear, correct, and unambiguous. Any typographical or grammatical errors should be corrected at revision, so please note any specific errors here.

Reviewer #1: Yes

Reviewer #2: Yes

**5. Review Comments to the Author**

Please use the space provided to explain your answers to the questions above. You may also include additional comments for the author, including concerns about dual publication, research ethics, or publication ethics. (Please upload your review as an attachment if it exceeds 20,000 characters)

| **Reviewer #1** | **Comment** | **Response** |
| --- | --- | --- |
| **Q(1)** | The relationship between RGB image indices and plant indices in tomato plants, along with regression analysis was investigated in this study. The two-year experimental period was involved and the experimental workload is quite heavy. Selection of image indices and plant indices were reasonable and the regression results were credible. However, several issues require clarification:  Sample data specifications: Providing sample size, open-field experimental area, and final tomato yield would enhance understanding of the experimental subjects. | Thank you for your valuable suggestion. The sample size is detailed in lines **181-197**, the open-field experimental area is specified in **line 108**, and the final tomato yield is presented in **lines 307-310**. |
| **Q(2)** | Methodological rigor: While the workload was substantial, the analytical approach lacks systematic completeness. For instance, in ANOVA, presenting both significance levels and effect size metrics (e.g., correlation coefficients) would help readers assess conclusion reliability and correlation strength between variables, rather than relying solely on significance levels. | Thank you for your comment. In response, we have added effect sizes to clarify the impact of irrigation regimes on FB, DB, CWC, SPAD, SMC, and yield. These values have been incorporated into the manuscript (**lines 261–307**). To determine significant differences between means, as presented in **Figure 3** and **Table 5**, Tukey’s post-hoc test was applied at significance levels of P ≤ 0.05, 0.01, and 0.001. Regression models were fitted to examine the relationships between plant traits and RGB image indices, as well as between actual and predicted values from the ANN models. The corresponding correlation coefficients are displayed in **Figures 4-7**. |
| **Q(3)** | Methodological innovation: Established models for image-plant index regression should be compared. Does the author's model demonstrate advantages over classical approaches or state-of-the-art methodologies? Comparative analysis is warranted to validate its innovation. | Thank you for your comment. The primary objective of this study was not to compare different modeling approaches, but rather to develop a non-destructive method for assessing plant characteristics using field and laboratory measurements as reference standards. Accordingly, the accuracy of the artificial neural network model was evaluated by comparing its predictions against actual field and laboratory values, as presented in **Tables 6-8**. This approach aligns with our goal of providing a reliable, time-saving alternative to conventional destructive methods that can also support irrigation management decisions. |
| **Reviewer #2** | **Comment** | **Response** |
| **Q(1)** | Manuscript is well organized. Material and methods are suitable for research purposes. This manuscript is acceptable for publication. There is one correction:  In line 119 CROWAT should be corrected as CROPWAT. | Thank you for your comment. The necessary adjustment has been made. |
