# Supplementary material for: Predicting water status, growth and yield of tomato under different irrigation regimes using the RGB image indices and artificial neural network model
Source: PLoS One. 2026 Apr 30;21(4):e0346503. doi: 10.1371/journal.pone.0346503 (PMC13132235; doi:10.1371/journal.pone.0346503)
Supplement: Attachment — Submitted filename: Comments-4.docx [file pone.0346503.s003.docx]

| **No.** | **Comments** | **Response** |
| --- | --- | --- |
| **1** | The irrigation model name is still incorrect in the manuscript text. Although the response letter states that this was corrected, the manuscript still reads “CROWAT” in the irrigation methods section. This should be corrected to “CROPWAT” consistently throughout. | We appreciate your comment and have revised the text accordingly. |
| **2** | Year / season information must be harmonized throughout the manuscript. The abstract refers to the 2021/2022 and 2022/2023 growing seasons, the Methods section states that the field experiments were conducted during 2022 and 2023, and the biomass sampling paragraph refers to measurements in 2021 and 2022. Please revise these sections so that the experimental timeline is fully consistent everywhere. | Thank you for your feedback. We have made the requested adjustments. |
| **3** | The ANN methodology requires clearer technical description. Please specify the actual correlation threshold used for feature selection, clarify exactly which scikit-learn implementation and solver(s) were used, and revise the description of training/testing versus cross-validation so that the workflow is unambiguous. At present, the method description remains difficult to follow in this respect. | We thank the reviewer for this comment. The ANN methodology has been revised as suggested (see lines 202-255). |
| **4** | Please check and correct the equation given for R². The reported formula appears incorrect/incomplete as presented in the current manuscript and should be verified carefully against the actual metric used in the analysis. | Thank you for your feedback. We have corrected the equation for R² as requested. |
| **5** | Please remove the remaining language and typographical errors throughout the manuscript. Examples visible in the revised version include “Re-mote,” “pcercentage,” “conent,” “soil moister,” “phybrid,” “Identify” instead of “Identity,” “These observed is,” and “DB wights.” Please perform a final careful proofreading of the full manuscript, tables, and figure legends. | We thank the reviewer for this feedback. As requested, we have corrected the remaining language and typographical errors throughout the manuscript. |
| **6** | Please revise the interpretation regarding T75 in the Discussion. The manuscript states that T75 was favorable “with no significant reduction in the tomato crop,” whereas the preceding results section states that Tukey post-hoc testing showed significant pairwise differences between treatments for yield. Please reconcile this statement with the actual statistical results and avoid overstating the conclusion. | We thank the reviewer for this feedback. We have revised the interpretation regarding T75 in the Discussion, as requested (see lines 343–346). |
|  |  |  |
